# An Evolved Strain of the Oleaginous Yeast *Rhodotorula toruloides*, Multi-Tolerant to the Major Inhibitors Present in Lignocellulosic Hydrolysates, Exhibits an Altered Cell Envelope

**DOI:** 10.3390/jof9111073

**Published:** 2023-11-02

**Authors:** Mónica A. Fernandes, Marta N. Mota, Nuno T. Faria, Isabel Sá-Correia

**Affiliations:** 1 iBB—Institute for Bioengineering and Biosciences, Instituto Superior Técnico, Universidade de Lisboa, Av. Rovisco Pais, 1, 1049-001 Lisbon, Portugal; 2 Department of Bioengineering, Instituto Superior Técnico, Universidade de Lisboa, Av. Rovisco Pais, 1, 1049-001 Lisbon, Portugal; 3 i4HB—Institute for Health and Bioeconomy, Instituto Superior Técnico, Universidade de Lisboa, Av. Rovisco Pais, 1, 1049-001 Lisbon, Portugal

**Keywords:** acetic acid, formic acid, 5-hydroxymethylfurfural (HMF), furfural, yeast cell wall, yeast cell permeability, non-conventional yeasts, lignocellulosic bioprocesses, microbial oils

## Abstract

The presence of toxic compounds in lignocellulosic hydrolysates (LCH) is among the main barriers affecting the efficiency of lignocellulose-based fermentation processes, in particular, to produce biofuels, hindering the production of intracellular lipids by oleaginous yeasts. These microbial oils are promising sustainable alternatives to vegetable oils for biodiesel production. In this study, we explored adaptive laboratory evolution (ALE), under methanol- and high glycerol concentration-induced selective pressures, to improve the robustness of a *Rhodotorula toruloides* strain, previously selected to produce lipids from sugar beet hydrolysates by completely using the major C (carbon) sources present. An evolved strain, multi-tolerant not only to methanol but to four major inhibitors present in LCH (acetic acid, formic acid, hydroxymethylfurfural, and furfural) was isolated and the mechanisms underlying such multi-tolerance were examined, at the cellular envelope level. Results indicate that the evolved multi-tolerant strain has a cell wall that is less susceptible to zymolyase and a decreased permeability, based on the propidium iodide fluorescent probe, in the absence or presence of those inhibitors. The improved performance of this multi-tolerant strain for lipid production from a synthetic lignocellulosic hydrolysate medium, supplemented with those inhibitors, was confirmed.

## 1. Introduction

The presence of toxic compounds in lignocellulosic hydrolysates (LCH) is among the main barriers affecting the efficiency of lignocellulose-based fermentation processes, in particular, to produce biofuels, hindering the production of intracellular lipids by oleaginous yeasts [1,2,3,4]. Microbial oils, produced by oleaginous yeasts, can reach up to 70% of dry cell weight [5,6,7] and are promising substitutes for vegetable oils for conversion into biodiesel [8,9,10]. The diverse growth and metabolism inhibitors present in lignocellulosic hydrolysates and the corresponding cultivation media belong to three major groups: weak acids, furan derivatives, and phenolic compounds arising from pretreatment, hydrolysis, and fermentation of lignocellulosic biomasses [1,11,12]. Organic acids originate from the breakdown of hemicellulose, phenolic compounds are breakdown products of lignin, and the furans furfural and 5-hydroxymethylfurfural (HMF) are derived from elimination reactions of pentose and hexose sugars, respectively [13,14]. The concentration of the inhibitors derived from diverse raw materials and resultant from different biomass pretreatments is quite variable [1,13,15]. In these lignocellulosic biomass hydrolysates, the concentration of acetic acid normally ranges from 1.0 to 15.0 g/L depending on the biomass feedstock and pretreatment methods used [1,13,15,16,17], while formic acid concentration is usually below acetic acid concentration. Furfural concentrations usually vary from 0.5 to 3.0 g/L and HMF from 2.0 to 5.9 g/L [1,13,15,16,17]. Due to their toxicity, they reduce microbial lipid productivity and yield as the result of the inhibition of important cellular processes and the diversion of energy into cellular adaptive responses to their deleterious effects [18]. In general, the inhibitory potential of the diverse compounds is quantified individually, but their combination may pose a great challenge to yeast cell factories. The detoxification of the hydrolysates involves expensive methods that also can reduce sugar content while the development of superior strains, with reduced susceptibility to the effects of LCH inhibitors, is an economically favorable strategy [19].

Although *Saccharomyces cerevisiae* is an essential experimental model yeast widely employed in biotechnology, natural strains lack many desired traits to be useful for a circular bioeconomy. In particular, they lack the capacity to use a wide range of carbon (C) sources, special metabolic potential, and high multi-stress tolerance. Therefore, the interest in non-*Saccharomyces* yeasts, like it is the case of basidiomycetes species of the *Rhodotorula* genus, is gaining momentum [16,20,21,22]. *R. toruloides* is an oleaginous red yeast species that is attracting great interest due to its ability to use the main C sources present in lignocellulosic hydrolysates (glucose, xylose, and acetic acid) to efficiently produce lipids and carotenoids [2,22,23,24,25]. Moreover, this species can also efficiently use other less usual and difficult-to-catabolize carbon sources, as is the case of the acid sugar *D*-galacturonic acid and the neutral sugar *L*-arabinose, present in sugar beet pulp hydrolysates and hydrolysates from other pectin-rich agro-industrial residues [23]. A robust *R. toruloides* strain was previously selected in our laboratory for sugar beet pulp valorization through the production of lipids and carotenoids by the complete catabolism of the major C sources present [23]. However, the metabolism of this promising strain IST536 (PYCC 5615), can be limited by the presence of acetic acid in the hydrolysates since this weak acid has a dual role in the bioprocess: it can be easily metabolized as carbon and energy source but, depending on the concentration and medium pH, can also be a metabolism inhibitor [23]. The first aim of this work was to increase the IST536 strain robustness to improve yeast performance in bioprocesses based on lignocellulosic hydrolysates; in this study, we explored adaptive laboratory evolution (ALE). This method of evolutionary engineering is simple and effective in improving yeast tolerance to increasing levels of selective pressure during extended cultivation for several generations [26,27]. In this case, the selective pressure was posed by inhibitory concentrations of methanol in a growth medium with glycerol as the carbon source at a concentration of 5% (*v*/*v*) that imposed an additional osmotic stress. The selected evolved strain was found to exhibit increased tolerance to methanol and, in this study, was also proved to be multi-tolerant exhibiting increased tolerance to the four main inhibitors present in lignocellulosic hydrolysates: acetic and formic acids, HMF, and furfural. Given the results, the second objective of this work was to obtain some mechanistic insights underlying such multi-tolerance, at the level of the cellular envelope. The better performance of this evolved multi-tolerant strain for lipid production from a synthetic lignocellulosic hydrolysate supplemented with those inhibitors was confirmed.

## 2. Materials and Methods

### 2.1. Yeast Strains

The strain *Rhodotorula toruloides* IST536 (PYCC 5615) and the mutant *R. toruloides* IST536 MM15, obtained during this work through an Adaptive Laboratory Evolution (ALE) experiment, as described in the Results Section, were used in this study. The strains were maintained at 4 °C in YPD (Table 1) agar plates. For long-term storage, the strains are preserved at −80 °C in YPD medium supplemented with 15% glycerol (*v*/*v*).

### 2.2. Culture Media and Culture Conditions

A rich YPD medium, a minimal medium (MM) and a synthetic lignocellulosic hydrolysate (SH) were used in this work. The MMGly was only used for the ALE experiments, supplemented with methanol (VWR, Darmstadt, Germany), with concentrations ranging from 5.0 to 6.6% (*v*/*v*) (Table 1). The minimal medium (MM) and the synthetic hydrolysate medium (SH) were filter sterilized using a 0.2 µm filter (Whatman^®^ Puradisc, Maidstone, UK).

All pre-cultures were started from yeast colonies grown on YPD plates and run in 100 mL shake flasks containing 50 mL of medium, incubated at 30 °C, for 24 h with orbital shaking (250 rpm).

For main yeast cultivation, pre-cultured cells were harvested by centrifugation at 4600× *g* for 5 min at 4 °C, washed twice in sterile water and then inoculated in 20 mL of the medium used in 100 mL shake flasks, at an initial OD_600nm_ of 1, unless stated otherwise. Yeast growth was performed at 30 °C with orbital agitation (250 rpm) and monitored by measuring the optical density at λ = 600 nm (OD_600nm_) using a U-2000 HITACHI spectrophotometer (Tokyo, Japan). When required, culture samples were periodically collected to determine the extracellular concentration of carbon sources used and the metabolites produced.

### 2.3. Isolation of a Multi-Tolerant Strain, Derived from R. toruloides IST536, by Adaptive Laboratory Evolution (ALE)

*R. toruloides* strain IST536 was cultivated for approximately 100 generations in minimal medium MMGly supplemented with increasing concentrations of methanol, starting from 5.0% (*v*/*v*) to 6.6% (*v*/*v*). A pre-culture grown in YPD medium overnight was used to inoculate (initial OD_600nm_ = 1) 20 mL of medium in a 100 mL flask. Cells were incubated at 30 °C and 250 rpm for 24 h. Thirty-one rounds of sub-culturing were carried out under increasing methanol concentrations, ensuring that a robust growth (OD_600nm_~10) was reached at the end of each passage. Samples from the fastest-growing culture in MMGly supplemented with 6.6% (*v*/*v*) methanol were plated on MMGly agar plates supplemented with 5.0 to 7.0% (*v*/*v*) methanol and incubated at 30 °C for 48 h. The colonies that visually appear to include cells more tolerant to methanol were re-streaked in MMGly supplemented with methanol concentrations ranging from 7.0 to 9.0% (*v*/*v*) to confirm phenotype stability. About 15 colonies were tested for growth in liquid MMGly supplemented with 6.6% (*v*/*v*) methanol, and the best-performing strain under this stress (IST 536 MM15) was selected.

### 2.4. Determination of the Concentration of Sugars and Organic Acids

Glucose, xylose and acetic acid were quantified using high-performance liquid chromatography (HPLC) (Hitachi LaChrom Elite, Tokyo, Japan). The culture samples collected were centrifuged in a microcentrifuge at 9700× *g* for 3 min, and 100 microliters of the supernatant were pipetted into vials and diluted in 900 microliters of 5 mM H_2_SO_4_. The concentration of carbon sources present in each sample was determined by HPLC (Hitachi LaChrom Elite, Tokyo, Japan), using a column Aminex HPX87H (Bio-Rad, Hercules, CA, USA) coupled with an ultraviolet (UV)/visible detector (for acetic acid detection) and refractive index detector (for glucose and xylose detection). Ten microliters of the sample were loaded in the column with 5 mM H_2_SO_4_ as mobile phase at a flow rate of 0.6 mL/min for 30 min. The column temperature was defined at 65 °C, and the refractive index detector at 40 °C. Concentrations were calculated using calibration curves prepared for each compound.

### 2.5. Assessment of Lipid Production by Nile Red Staining

The assessment of lipid production was carried out based on Nile Red staining [28]. Cells were collected by centrifugation (2400× *g*, 3 min) to a final OD_600nm_ of 1.5. The pellet was resuspended and washed twice with a solution of 0.9% (*w*/*v*) NaCl. After the washing steps, pellets were resuspended in Phosphate-Buffered Saline (PBS; 1X, pH 7.4, containing 8 g/L NaCl (Sigma, Missouri, MO, USA), 0.2 g/L KCl (Sigma, Missouri, MO, USA), 1.44 g/L Na_2_HPO_4_ (Sigma, Missouri, MO, USA) and 0.24 g/L KH_2_PO_4_ (Sigma, Missouri, MO, USA)) and incubated at 50 °C for 30 min. A total of 200 microliters of the cell suspension were transferred to a black 96-well clear bottom plate (Thermo Fisher Scientific, New York, NY, USA), to measure the cell optical density at 595 nm in a FilterMax F5 Multi-Mode Microplate Reader (Molecular Devices, Sunnyvale, CA, USA). Cell suspensions were stained with Nile Red at a final concentration of 2.5 µg/mL and the relative fluorescence units (RFU) were measured using a FilterMax F5 Multi-Mode Microplate Reader (Sunnyvale, CA, USA) using the excitation and emission wavelengths of 535 and 625 nm, respectively. To obtain the final values of relative fluorescence units, the background fluorescence values were subtracted: (i) cells with PBS without Nile Red to eliminate buffer autofluorescence, and (ii) PBS with Nile Red. A normalization step of fluorescence to OD_595nm_ was also performed to consider the variation in cell concentration.

### 2.6. Cell Lipid Content and Lipid Profile by Gas Chromatography

The lipids present in freeze-dried cells obtained by centrifugation of cultures (4600× *g*, 5 min) were subjected to a methanolysis for the determination of the fatty acid profiles. Pure methanol (20 mL) was cooled to 0 °C, and 1 mL of acetyl chloride was added to create a water-free HCl/methanol solution. Culture broth samples (1 mL) were freeze dried, weighed, and then mixed with 2 mL of the HCl/methanol solution. This mixture was incubated for 1 h at 80 °C to convert the lipids into methyl esters, using heptanoic acid as an internal standard. The resulting product was extracted with hexane (1 mL), and 1 μL of the organic phase was injected into a gas chromatography (GC) system (Hewlett-Packard, HP5890, Wilmington, DE, USA). The GC system was equipped with an FID detector and an Agilent HP Ultra2 capillary column (length 50 m × inner diameter 0.32 mm, film thickness 0.52 μm). The oven temperature was programmed as follows: an initial temperature of 140 °C, followed by three temperature gradients—140 to 170 °C at a rate of 15 °C/min, 170 to 210 °C at 40 °C/min, and 210 to 310 °C at 50 °C/min. A final hold time of 3 min at 310 °C was defined. Carrier gas was used with a 1/25 split ratio.

### 2.7. Comparison of Yeast Tolerance to Inhibitors Present in the Lignocellulosic Hydrolysates

The tolerance of the selected evolved strain (*R. toruloides* IST536 MM15) to acetic acid, formic acid, furfural, and HMF, was compared with the original strain (*R. toruloides* IST536) based on the growth curves in MM medium supplemented with increasing concentrations of the inhibitors. The following concentrations were used: for acetic acid, 50 mM (3.00 g/L), 55 mM (3.30 g/L), and 60 mM (3.60 g/L); for formic acid, 40 mM (1.84 g/L), 50 mM (2.30 g/L), and 55 mM (2.53 g/L); for furfural 1.50 g/L, 2.00 g/L, and 3.00 g/L; and for HMF, 3.00 g/L, 5.00 g/L, and 7.00 g/L. Growth was followed by measuring optical density at λ = 600 nm (OD_600nm_), during 80 h of cultivation.

### 2.8. Yeast Cell Wall Susceptibility to Zymolyase

To monitor eventual yeast cell wall structural alterations, cells of the original and evolved strains pre-grown in YPD were cultivated for 1 h in MM medium supplemented or not with (i) 50 mM/3.00 g/L acetic acid; (ii) 40 mM/1.84 g/L formic acid; (iii) 1.50 g/L/15.61 mM furfural; and (iv) 3.00 g/L/23.79 mM HMF. Cells were harvested by centrifugation in a benchtop centrifuge (4600× *g*, 5 min) after 1 h of incubation, washed with distilled water, and used to inoculate 100 mL shake flasks containing 20 mL of 10 mM Tris HCL buffer (pH 7.5) to a final standardized OD_600nm_ of 1.0. Zymolyase susceptibility assay, using β-1,3-glucanase from *Arthrobacter luteus*, (Zymo Research, Irvine, CA, USA, batch ZRC164631), containing β-(1→3)-glucan laminaripentaohydrolase and β-(1→3)-glucanase activities), was conducted as described before [29,30]. After the addition of 2.5 U/mL of zymolyase, cell lysis was followed by measuring the decrease in OD_600nm_ for each cell suspension every 15 min for 2 h. Values were converted into a percentage of the initial OD_600nm_ value. The susceptibility to zymolyase is represented as the maximum specific lysis rate defined as the absolute value of the slope of the straight line that best fits the semi-logarithmic plot of the linear part of the lysis curve.

### 2.9. Assessment of Yeast Plasma Membrane Permeability Based on Propidium Iodide Fluorescent Probe

Plasma membrane permeability was assessed by propidium iodide (PI, Sigma) staining, essentially as described before with few modifications [31], using flow cytometry (BD Accuri™ C6 Plus (BD Biosciences, San Jose, CA, USA), to obtain Relative Fluorescence Units (RFUs).

Yeast cells pre-grown in YPD and then cultivated for 1 h in MM medium supplemented with the concentrations of inhibitors described in Section 2.7 were harvested and resuspended to an OD_600nm_ of 1.2 in PBS, as described for experiments of cell wall lysis. Cells were stained for 10 min with 10 μg/mL PI at 30 °C with orbital agitation (100 rpm). The PI fluorescence was collected in the flow cytometer via a FL2 585/40 nm filter. A sample of unstained cells was used to define the cell population (R1). As a positive control for cells with maximum permeability, a sample of cells was incubated with absolute ethanol at 30 °C for 15 min, centrifuged to remove ethanol, and stained with PI as described. The R1 population was then divided into two sub-populations. Cells grown in unstressed conditions and stained with PI were used to define a sub-population (M1) and cells treated with ethanol and PI were used to define a complementary sub-population (M2) of PI-positive cells. A total of 50,000 events per sample in M1 were acquired using a slow flow rate (14 μL/min).

## 3. Results

### 3.1. Adaptive Laboratory Evolution (ALE) of IST 536

In the framework of another project aiming to obtain a *R. toruloides* methanol-tolerant strain, *R. toruloides* IST536 was cultivated for approximately 100 generations (corresponding to thirty-one rounds of sub-culturing) in minimal medium MMGly supplemented with increasing concentrations of methanol, starting from 5.0% (*v*/*v*) to 6.6% (*v*/*v*), as described in the Materials and Methods Section. Samples from the fastest-growing culture in MMGly supplemented with 6.6% (*v*/*v*) methanol were plated on MMGly agar medium plates supplemented with 5.0 to 7.0% (*v*/*v*) methanol, and the colonies that appear visually to include cells more tolerant to methanol were re-streaked in MMGly supplemented with higher methanol concentrations [from 7.0 to 9.0% (*v*/*v*)] to ensure the stability of the phenotype. The best-performing strain under this stress (IST 536 MM15) was selected and examined in this work, for increased tolerance to different toxic compounds present in the hydrolysates: acetic and formic acids, HMF, and furfural.

### 3.2. The Evolved Strain R. toruloides IST536 MM15 Is More Tolerant to Multiple Inhibitors, Compared with the Original Strain

**Figure 1:**
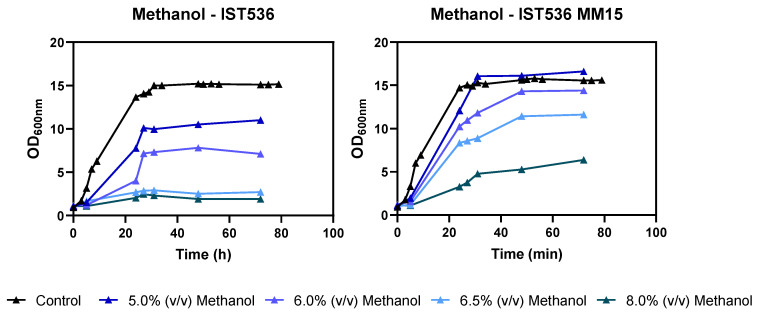
Effect of increasing concentrations of methanol in *R. toruloides* IST536 and *R. toruloides* IST536 MM15 growth curves (linear scale) when cultured in MM medium, at pH 4.5.

The evolved strain obtained by ALE, IST 536 MM15, was confirmed in MM medium as being more tolerant to inhibitory concentrations of methanol, compared with the original strain (Figure 1).

**Figure 2:**
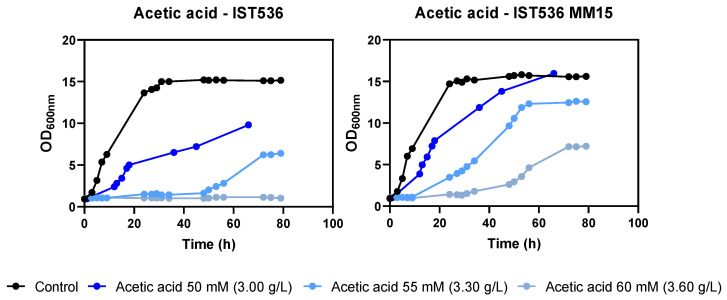
Effect of increasing concentrations of acetic acid in *R. toruloides* IST536 and *R. toruloides* IST536 MM15 growth curves (linear scale) when cultured in MM medium at pH 4.5.

**Figure 3:**
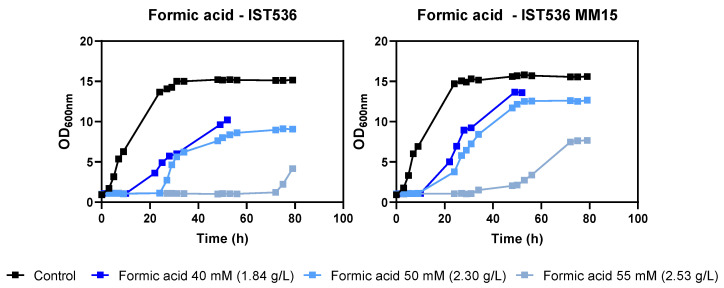
Effect of increasing concentrations of formic acid in *R. toruloides* IST536 and *R. toruloides* IST536 MM15 growth curves (linear scale) when cultured in MM medium at pH 4.5.

**Figure 4:**
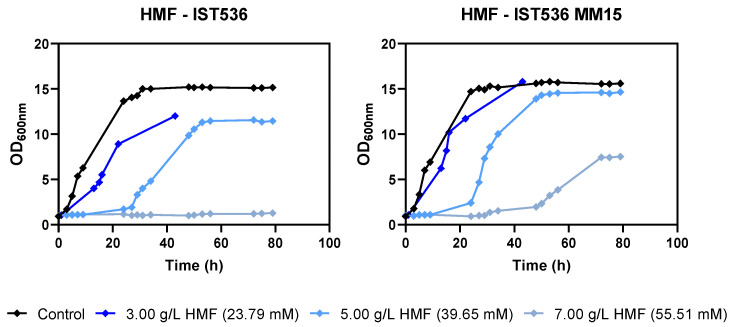
Effect of increasing concentrations of HMF in *R. toruloides* IST536 and *R. toruloides* IST536 MM15 growth curves (linear scale) when cultured in MM medium at pH 4.5.

**Figure 5:**
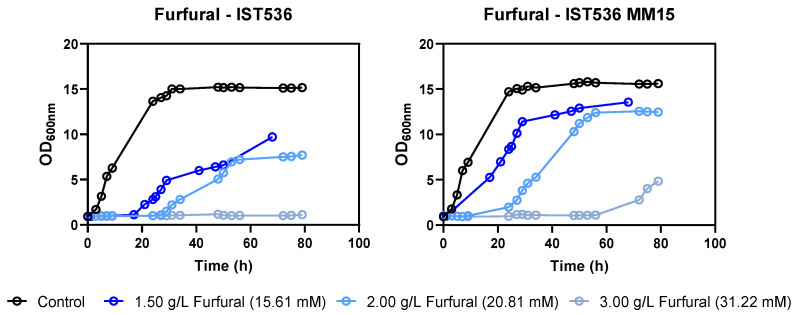
Effect of increasing concentrations of furfural in *R. toruloides* IST536 and *R. toruloides* IST536 MM15 growth curves (linear scale) when cultured in a MM medium at pH of 4.5.

The tolerance of this methanol-tolerant mutant towards growth inhibitors present in lignocellulosic biomass hydrolysates was tested. This evolved strain was found to be also more tolerant to acetic acid, formic acid, HMF and furfural by comparing the growth curves in MM medium at a pH of 4.5 (Figure 2, Figure 3, Figure 4 and Figure 5). This low pH was chosen because it is closer to the pKa of the weak acids to be tested (pKa 3.75 for formic acid and 4.75 for acetic acid), leading to higher toxicity. This simple and well-defined MM medium with glucose as the carbon source was chosen to avoid other sugar metabolic interferences in tolerance assessment [32].

Depending on the concentration and the inhibitor tested, this may be reflected in a decreased duration of growth latency, a higher maximum specific growth rate and a higher biomass concentration at the stationary phase for the evolved strain, compared with the original strain, under the same level of stress. Regardless of the strain, furfural was the compound with a stronger inhibitory effect for the same molar concentration tested, followed by HMF (Figure 5).

**Figure 6:**
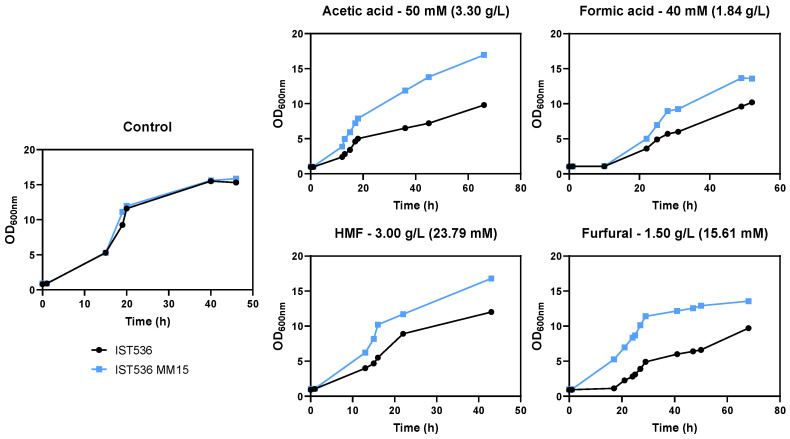
Effect of moderate equivalent concentrations of acetic acid, formic acid, HMF and furfural in the growth curves of *R. toruloides* IST536 (black curves) and *R. toruloides* IST536 MM15 (blue curves) when cultured in MM medium at pH 4.5. The concentrations of the inhibitors added to the growth medium are indicated above each panel.

To obtain insights into the mechanisms underlying the higher multi-tolerance exhibited by IST536 MM15, compared with the original strain, moderate equivalent concentrations of the four inhibitors were chosen to compare the performance of the evolved strain versus the original one (Figure 6). Growth curves in Figure 6 confirm former results and these concentrations were used to cultivate the cells to be used to compare important characteristics of the cellular envelope of both strains.

### 3.3. The Susceptibility of the Evolved Multi-Tolerant R. toruloides Strain to Zymolyase Activity Is below the Original Strain Susceptibility in Absence or Presence of Inhibitors

**Figure 7:**
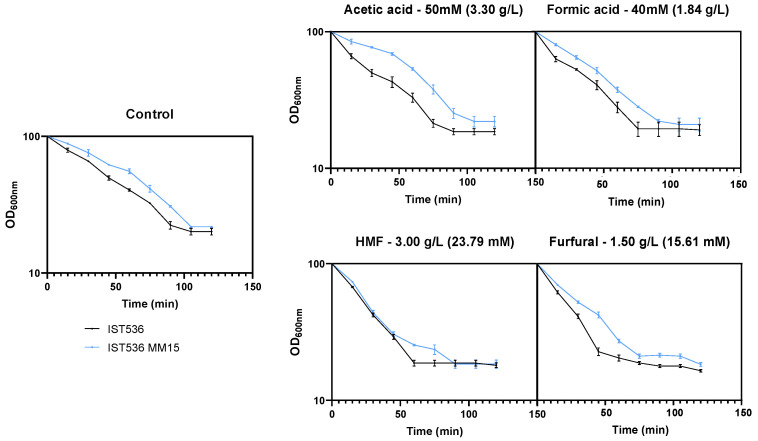
Effect of zymolyase in the cell wall of *R. toruloides* IST536 and *R. toruloides* IST536 MM15 after 1 h of incubation in MM medium in the absence or following sudden addition of the different inhibitors at the concentrations indicated above each panel. Cells used in this experiment were taken after 1 h of cultivation as shown in the growth curves in Figure 6. The decrease in the OD_600nm_ of cell suspensions (in percent) was measured following the addition of zymolyase, as described in M&M. Data are means from at least three independent experiments and bars represent standard deviation.

Considering the multi-tolerance of the evolved strain, it was hypothesized that the physicochemical properties of the cell wall could underlie such robustness, as highlighted before [29,33]. To test this hypothesis, we compared the susceptibility of the cell wall of the evolved strain with the original strain to zymolyase, a lytic enzyme, prepared from *Arthrobacter luteus*, and described as having a powerful effect in the lysing of viable yeast cells [34]. Zymolyase was previously found to be capable of degrading the cell wall of several yeast species, in particular *Rhodotorula* species [35], which was confirmed in our study (Figure 7). The cells to be compared were cultivated in MM medium for 1 h, in the absence or sudden presence of each inhibitor, at equivalent inhibitory concentrations, as shown in Figure 6.

**Figure 8:**
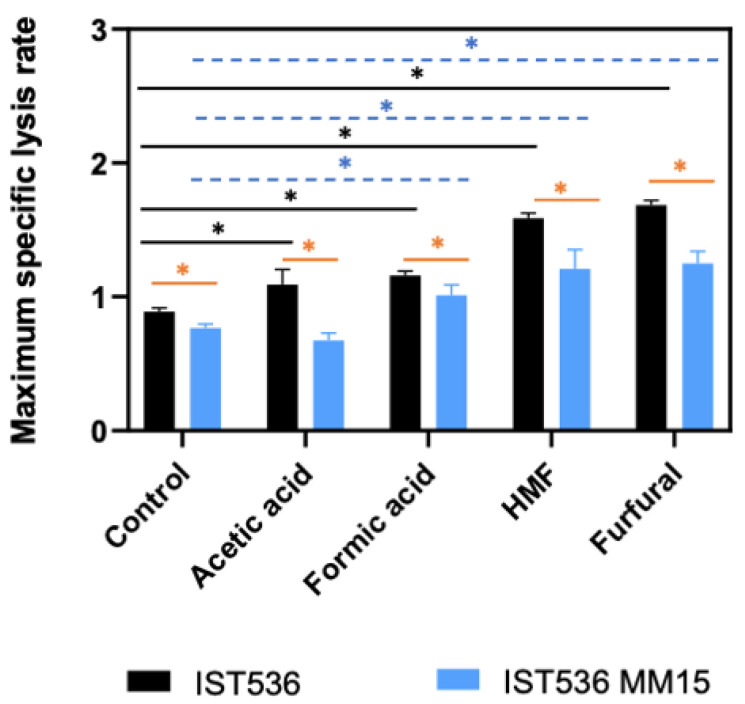
Maximum specific cell wall lysis rates determined based on the data in Figure 7, as described in M&M. The data shown represents means of three independent experiments, and the error bars indicate standard deviation. Asterisks indicate statistically significant different values (*p* < 0.05; *t*-student test).

Cells grown in minimal medium MM, in the absence or presence of inhibitors, exhibited differences in their susceptibility to zymolyase with the evolved mutant cells being significantly less susceptible to zymolyase activity than the original strain (Figure 7 and Figure 8). The maximum specific lysis rate for the cell wall of both strains cultivated in the presence or the absence of each inhibitor was calculated as the slope of the trend line that best fits the linear part of the semi-logarithmic plot of the decrease in cell suspension OD_600nm_ following the addition of zymolyase (Figure 8). The higher maximum specific lysis rate values calculated for the original strain compared with the evolved strain are statistically significant (*p* < 0.05; *t*-student test) in the absence of presence of inhibitors. This experimental evidence indicates that there are differences in the physicochemical properties of the respective cell walls. In general, cells exposed, for a short period of time (1 h), to the different toxicants tested also exhibited altered profiles of zymolyase susceptibility, compared with the non-stressed cells. However, the evolved strain exhibited similar results, compared with the original strain, when cultivated in the absence of inhibitors, with the evolved strain evidencing a significantly less susceptible cell wall, independently of the inhibitor present in the cultivation medium (Figure 7 and Figure 8). In the case of HMF or furfural, a marked increase in cell wall susceptibility to zymolyase of both strains was observed following cultivation for 1 h with those inhibitors (Figure 7), suggesting a significant deleterious effect of furfural and HMF at the cell wall level. Cultivation with acetic and formic acids under identical conditions led to a less marked increase in cell wall susceptibility, with the exception of the evolved strain. This different behavior could be due to the high acetic acid tolerance trait of the evolved strain. Since the experimental approach used in these experiments only examined one time of cultivation with the different inhibitors, it is likely that this evolved strain was able to, more rapidly and efficiently, induce the necessary adaptive responses [26].

### 3.4. The Evolved Multi-Tolerant R. toruloides Strain Permeability to Propidium Iodide, Is below the Original Strain Permeability in Absence or Presence of Inhibitors

**Figure 9:**
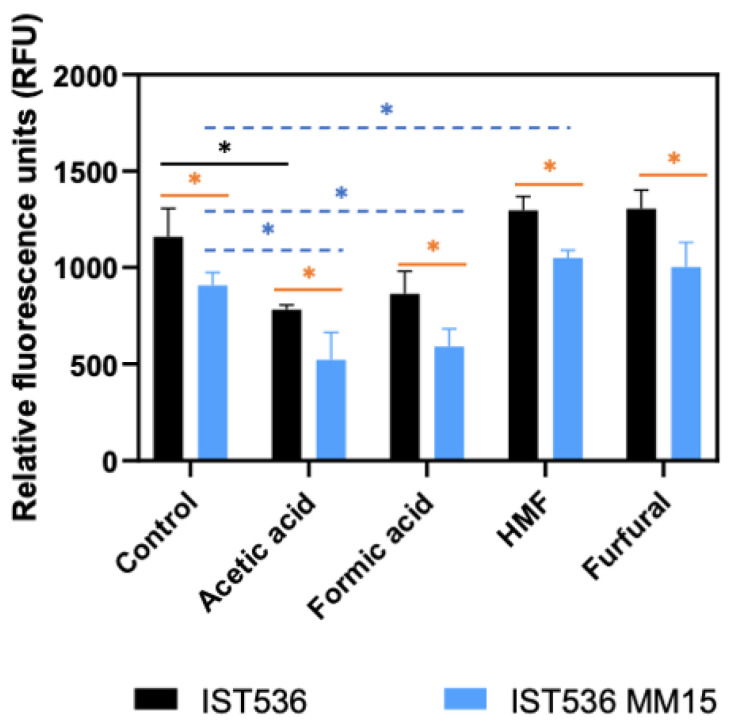
Permeability of cells of *R. toruloides* IST536 and *R. toruloides* IST536 MM15 obtained by flow cytometry analysis using propidium iodide fluorescent probe. Plasma membrane permeability was compared using cells incubated for 1 h in the MM medium with and without the inhibitors, as for the cell wall susceptibility experiments. The data shown represents means of three independent experiments, and the error bars indicate standard deviation. Asterisks indicate statistically significant different values (*p* < 0.05; *t*-student test).

Yeast cell permeability was assessed by flow cytometry analysis of cells incubated with the propidium iodide (PI) fluorescent probe. These cells were cultured in the same medium and for the same short period of time used to assess cell wall susceptibility to zymolyase. The original strain cells were used as PI-negative control, and these cells permeabilized by contact with absolute ethanol for 15 min were used as a PI-positive control. The permeability of the original and the evolved strains were compared in the absence or presence of the inhibitors under study (Figure 9).

The higher permeability of cells of the original strain compared with the permeability of the evolved strain, in the absence or presence of inhibitors, is statistically significant (*p* < 0.05; *t*-student test). Moreover, the relation of the permeabilities of both strains cultivated with or without the inhibitors for 1 h is, in general, similar to the relation shown above for cell susceptibility to zymolyase, with the original strain being significantly more permeable than the evolved strain. Concerning the eventual alteration of plasma membrane permeability as the result of cultivation for one hour with each inhibitor, most of the alterations observed indicate a less permeable membrane and were observed for the evolved strain for most of the inhibitors, with the exception of furfural, suggesting the occurrence of an adaptive response, as reported before for acetic acid [29]. Concerning the original strain, the sole significant difference was observed for acetic and was also suggestive of the occurrence of an adaptive mechanism leading to membrane impermeabilization.

### 3.5. The Evolved R. toruloides Strain Performed Better in Synthetic Medium Simulating a Lignocellulosic Hydrolysate, Compared with the Original Strain

**Figure 10:**
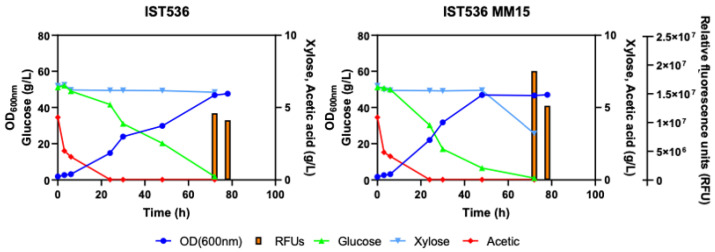
Growth curves, carbon source utilization and lipid production by *R. toruloides* IST536 and *R. toruloides* IST536 MM15 cultivated in a synthetic medium simulating a lignocellulosic hydrolysate (SH) at initial pH 5.5. Lipid production was assessed by Nile Red staining and is shown as relative fluorescence units (RFU). Results are from a representative experiment of other independent experiments leading to similar results.

Given the higher multi-tolerance of the evolved *R. toruloides* strain to the four major inhibitors present in lignocellulosic hydrolysates, the performance of this evolved strain concerning lignocellulosic hydrolysate-based lipid production was compared with the performance of the original strain (Figure 10). A synthetic lignocellulosic hydrolysate medium (medium SH, described in Table 1) including acetic and formic acids as well as furfural and HMF, at concentrations in the range of those reported in the literature [13,36,37] was tested. The initial pH of the medium was set to 5.5 to reduce the toxicity of acetic acid present at significant concentrations [38,39].

Under the experimental conditions used, the evolved multi-tolerant strain was completely used at a faster utilization rate compared with the original strain, the acetic acid and glucose present in the medium. However, xylose that is only used by *R. toruloides* after glucose exhaustion, due to catabolic repression, was not totally consumed even after 80 h of cultivation, suggesting that the stationary phase was attained due to other culture limitations, presumably due to the decrease in external pH and accumulation of organic acids since there was no pH control or fine optimization of other bioprocess conditions [23,40,41]. In the case of the original strain, after 80 h of incubation, xylose was still unused, consistent with the slower consumption rate of glucose which was only exhausted by that time. Acetic acid (5 g/L) was the first carbon source to be consumed by both strains, followed by glucose (60 g/L), as described before [23]. Both strains were able to co-consume acetic acid and glucose posing an advantage from an industrial point of view, once both carbon sources are present, as is the case of lignocellulosic hydrolysates [23]. Lipid production, assessed based on the Nile Red method, reached its maximum at the early stationary phase (72 h) for both strains. Lipid quantification was also performed using Gas Chromatography. Results indicate that the evolved multi-tolerant strain is also a better lipid producer concerning total lipid content (24.9% of dry cell biomass for IST 536 compared with 36.2% of dry cell biomass for the evolved strain). The profiles of the different fatty acids present in cells harvested after 72 h of cultivation are shown in Table 2. Results indicate that, likely due to the higher specific lipid production rate for the evolved strain, compared with the original strain, the percentage of shorter chain fatty acids is lower for the evolved strain while the percentage of C18:1 was higher, reaching 56% of the dry biomass compared with 41% for the original strain.

## 4. Discussion

This study illustrates the applicability of the Adaptive Laboratory Evolution (ALE) strategy as a powerful tool to improve the performance of a non-conventional yeast species of biotechnological relevance, specifically, to enhance *Rhodotorula toruloides* tolerance to four major growth inhibitors present in lignocellulosic biomass hydrolysates. As found in this work, the ALE approach allows the increase in yeast genetic and phenotypic diversity and robustness without artificial manipulation of the genome [26,27,42], leading to faster growth rates, higher biomass production and the ability to efficiently consume the sugars present in agro-forest-derived feedstocks coupled to enhanced bioproduct formation [43,44]. The ALE experiment carried out in this study was based on serial batch cultivation, which is the simplest and most popular ALE procedure, frequently employed. The selective conditions used in the ALE experiment, specifically, the presence of increasing methanol inhibitory concentrations in a growth medium with glycerol as a carbon source at a concentration that imposed some osmotic stress, were applied for approximately 100 generations, corresponding to a selection period of approximately three consecutive months. The use of methanol supplementation of a minimal medium with glycerol as a carbon source to impose selective pressures to obtain this multi-tolerant strain was merely casual and independent of the main objective of the current work. It was due to our interest in developing a more methanol-tolerant strain capable of growing efficiently in the referred growth medium. The observation that this strain was not only more tolerant to methanol but also to acetic acid, formic acid, furfural and HMF, is considered a highly interesting trait to be explored in biotechnology but also to provide further clues into the multi-resistance phenomenon.

Results obtained in this study clearly demonstrate the multi-tolerance of the evolved strain to these four chemical stresses (acetic acid, formic acid, HMF, and furfural) when compared with the original strain. Furfural, followed by HMF, led to higher growth inhibitory effects for the same molar concentration. Given the higher multi-tolerance of the evolved strain, compared with the original strain, the analysis of the characteristics of the cell envelope, due to its major role as the first line of defense against a wide range of environmental stresses [33], was considered. In fact, cell envelope composition and biophysical properties and its remodeling under stress are among the mechanisms behind yeast adaptation and tolerance to multiple stresses [16,45]. Results confirm our work hypothesis and the susceptibility of the evolved strain wall to zymolyase activity was below the original strain susceptibility. This was observed in cells cultivated either in the absence or presence of inhibitors. For the short incubation time (1 h), stressed cells became more susceptible to enzymatic lysis indicating the deleterious action of these chemical stresses. The sole exception was registered for the more tolerant strain challenge with acetic acid suggesting that it was able to induce an effective adaptive response to this stress in this short time. Changes in cell wall biophysical properties, such as the stiffness of the cell surface, mostly dependent on the cross-linking between β-glucans and chitin [46], have been reported to occur in response to industrially relevant stresses [47,48,49]. A complex time course of these alterations depends on strain tolerance, level of stress and phase of growth [47]. The activation of the cell wall integrity pathway modulates the expression of specific genes related to cell wall biogenesis [50]. There are examples of successful alterations of the physicochemical properties of the yeast cell wall either by ALE or by genetic engineering impacting yeast tolerance to stress(es) [33]. The important role played by the cell wall in yeast adaptation and tolerance to different stresses of biotechnological interest clearly emerges from a recent review article [51]. Results here described are in line with those reported in the scientific literature on the topic that involve the cell wall, as a dynamic organelle, in cross-stress protection [51]. However, most of the results on the topic reported so far have been dedicated to the model yeast and cell factory *Saccharomyces cerevisiae* being scarce and the available information concerning non-conventional yeast species of biotechnological relevance. Also for this reason, results obtained in this work for the basidiomycete yeast species *R. toruloides* are an important addition to current knowledge in the field.

Results from the present study also indicate that the evolved strain has a reduced cellular permeability, in particular a reduced non-specific plasma membrane permeability, compared with the original strain, following 1 h of incubation in the absence or presence of inhibitors. Plasma membrane composition and remodeling under stress are also considered essential for stress tolerance in yeast. In the particular case of acetic acid, the adequate incorporation of sphingolipids and ergosterol in the plasma membrane leads to decreased plasma membrane non-specific permeabilization under acetic acid stress, both in *Saccharomyces cerevisiae* and in the highly tolerant food spoilage yeast species *Zygosaccharomyces bailii* [52,53,54,55]. Differently from what was observed when the same cells incubated for 1 h with the same inhibitor concentrations were examined concerning cell wall susceptibility to zymolyase, their permeability was not significantly increased under stress. On the contrary, with the exception of furfural, the evolved cells were less permeable following 1 h of adaptation to the inhibitors-induced stress. In the case of the less tolerant original strain, such adaptive indication was only significant for acetic acid. It has to be noted that either cell wall susceptibility to zymolyase or cellular permeability was only evaluated after one hour of incubation with the inhibitors. These results are consistent with another report showing that short-term adaptation of yeast strains may improve yeast membrane integrity [56]. Given the complex time course of plasma membrane and cell wall remodeling under stress [29,47], it is likely that a more extended incubation time could lead to the occurrence of more generalized adaptation mechanisms. For example, membrane remodeling mechanisms involving the increased production of ergosterol were also reported in HMF-stressed cells [57], contributing to the integrity of the cell membrane and physiological selective permeability [29,57].

Collectively, our results suggest that the multi-tolerant phenotype of the evolved strain is, at least partially, the result of a more protective cellular envelope against diverse toxic chemicals present in the external medium. These results are consistent with the notion that there is a crosstalk between the plasma membrane and cell wall biophysical properties, suggesting a coordinated response to counteract the deleterious effects of chemical stresses [33]. These responses guarantee a robust adaptive response. In the particular case of the weak acids acetic and formic acids, this response is essential to limit the futile cycle associated with the re-entry of the acid form after the active expulsion of the counterion from the cell interior [47].

This work can be considered a proof of concept concerning the development by ALE of an efficient oleaginous yeast strain multi-tolerant to four major inhibitors present in lignocellulosic hydrolysates. The objective of the development of industrial strains is to maximize bioprocess productivity (product formation in unit time) and yield (product formation per substrate consumption). The exploitation of ALE to improve the biotechnological potential of nonconventional yeasts was confirmed as a promising strategy. given that the evolved strain exhibits a better performance concerning lipid productivity from a toxic synthetic lignocellulosic hydrolysate. Given that *R. toruloides* is capable of using a wide range of carbon sources, this evolved strain, more tolerant to the four major inhibitors present in lignocellulosic hydrolysates, represents an advantage in terms of the exploitation of promising lignocellulosic biomass feedstocks in bioprocesses envisaging the transition to a sustainable bio-based economy.

## Figures and Tables

**Table 1:** Composition of the basal culture media used in this study.

Designation and Short Description	Composition
Minimal medium with glucose (MM) Glycerol (MMGly, for ALE experiments)	6.7 g/L YNB (Difco, Michigan, MI, USA) with: 20.0 g/L glucose (NZYTech, Lisbon, Portugal) (MM) or 50.0 g/L of glycerol (Scharlau, Barcelona, Spain) (MMGly) pH adjusted to 4.5
Synthetic medium simulating lignocellulosic hydrolysate (SH)	1.3 g/L of YNB without ammonium sulfate and amino acids (Difco); supplemented with: 1 g/L de (NH_4_)_2_SO_4_ (Panreac, Chicago, IL, USA); amino acids (20 mg/L of L-histidine, 40 mg/L DL-methionine and 40 mg/L DL-tryptophan, all from Sigma, Darmstadt, Germany); 60.0 g/L glucose (NZYTech, Lisbon, Portugal); 7.0 g/L xylose (Sigma-Aldrich, Darmstadt, Germany); 5.0 g/L of acetic acid (Fluka, Buchs, Switzerland); 0.7 g/L of formic acid (Fluka, Buchs, Switzerland); 0.4 g/L of furfural (Sigma-Aldrich, Darmstadt, Germany); 1.0 g/L of HMF (Sigma-Aldrich, Darmstadt, Germany). pH adjusted to 5.5
Yeast peptone dextrose (YPD)	20.0 g/L of glucose (NZYTech, Lisbon, Portugal); 20.0 g/L of bactopeptone (Gibco, Detroit, MI, USA); 10.0 g/L of yeast extract (VWR, Darmstadt, Germany).

**Table 2:** Fatty acid composition of the lipids of IST 536 and IST 536 MM 15 after 72 h of cultivation at 30 °C, at pH 5.5.

	Fatty Acid Content (%, *w*/*w*)	
Fatty Acid Type	IST 536	IST 536 MM15
C14:0	2.3	1.8
C16:0	31.7	24.6
C16:1	0.8	0.9
C18:0	24.2	16.6
C18:1	41.0	56.1
Total lipid content	24.9	36.2

## Data Availability

Data sharing not applicable.
